# 2-[(Anilino)(2-nitro­phen­yl)meth­yl]cyclo­hexa­none

**DOI:** 10.1107/S1600536812036859

**Published:** 2012-09-01

**Authors:** Bagher Eftekhari-Sis, Sahar Mohajer, Orhan Büyükgüngör

**Affiliations:** a Department of Chemistry, University of Maragheh, Maragheh, Iran; b Department of Physics, Ondokuz Mayis University, TR-55139 Samsun, Turkey

## Abstract

In the title compound, C_19_H_20_N_2_O_3_, the cyclo­hexa­none ring adopts a chair conformation with the amino­methyl group is positioned equatorially. An intra­molecular N—H⋯O hydrogen bond occurs. In the crystal, mol­ecules are linked by N—H⋯O hydrogen bonds.

## Related literature

For the synthesis of the title compound and related compounds, see: Eftekhari-Sis *et al.* (2012*a* ▶,*b* ▶). For the biological activity of *β*-amino ketones, see: Arend *et al.* (1998 ▶). For the anti-inflammatory and anti­microbial activity of *β*-amino ketones, see: Jadhav *et al.* (2008 ▶) and Kalluraya *et al.* (2001 ▶), respectively. For information on the Mannich reaction, see: Eftekhari-Sis *et al.* (2006 ▶); Samet *et al.* (2009 ▶); Azizi *et al.* (2006 ▶); Cordova (2004 ▶). For related structures, see: Eftekhari-Sis *et al.* (2012*b* ▶); Yuan *et al.* (2007 ▶); Fun *et al.* (2009 ▶).
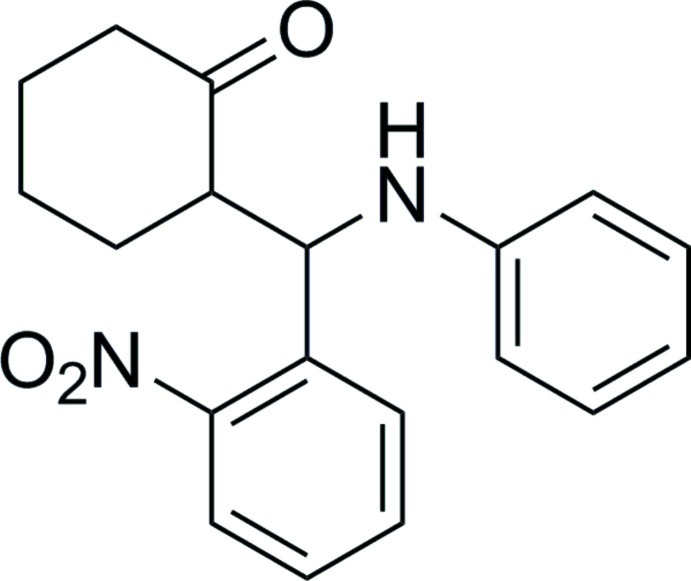


## Experimental

### 

#### Crystal data

C_19_H_20_N_2_O_3_
*M* *_r_* = 324.37
Monoclinic, 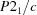

*a* = 9.0535 (8) Å
*b* = 11.9947 (7) Å
*c* = 17.2267 (15) Å
β = 117.355 (6)°
*V* = 1661.5 (2) Å^3^
*Z* = 4
Mo *K*α radiation
μ = 0.09 mm^−1^
*T* = 296 K
0.62 × 0.43 × 0.21 mm

#### Data collection

Stoe IPDS 2 diffractometer
Absorption correction: integration (*X-RED32*; Stoe & Cie, 2002 ▶) *T*_min_ = 0.784, *T*_max_ = 0.958
10888 measured reflections
3434 independent reflections
2210 reflections with *I* > 2σ(*I*)
*R*_int_ = 0.043

#### Refinement

*R*[*F* ^2^ > 2σ(*F* ^2^)] = 0.054
*wR*(*F* ^2^) = 0.160
*S* = 0.97
3434 reflections
221 parameters
16 restraints
H atoms treated by a mixture of independent and constrained refinement
Δρ_max_ = 0.79 e Å^−3^
Δρ_min_ = −0.23 e Å^−3^

### 

Data collection: *X-AREA* (Stoe & Cie, 2002 ▶); cell refinement: *X-AREA*; data reduction: *X-RED32* (Stoe & Cie, 2002 ▶); program(s) used to solve structure: *SHELXS97* (Sheldrick, 2008 ▶); program(s) used to refine structure: *SHELXL97* (Sheldrick, 2–8); molecular graphics: *ORTEP-3 for Windows* (Farrugia, 2012 ▶); software used to prepare material for publication: *WinGX* (Farrugia, 2012 ▶).

## Supplementary Material

Crystal structure: contains datablock(s) I, global. DOI: 10.1107/S1600536812036859/fj2593sup1.cif

Structure factors: contains datablock(s) I. DOI: 10.1107/S1600536812036859/fj2593Isup2.hkl

Supplementary material file. DOI: 10.1107/S1600536812036859/fj2593Isup3.mol

Supplementary material file. DOI: 10.1107/S1600536812036859/fj2593Isup4.cml

Additional supplementary materials:  crystallographic information; 3D view; checkCIF report

## Figures and Tables

**Table 1:** Hydrogen-bond geometry (Å, °)

*D*—H⋯*A*	*D*—H	H⋯*A*	*D*⋯*A*	*D*—H⋯*A*
N1—H1*A*⋯O1	0.85 (2)	2.31 (2)	2.906 (3)	127.0 (18)
N1—H1*A*⋯O2^i^	0.85 (2)	2.48 (2)	3.246 (3)	150.2 (19)

Symmetry code: (i) 
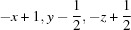
.

**Fig. 1.**
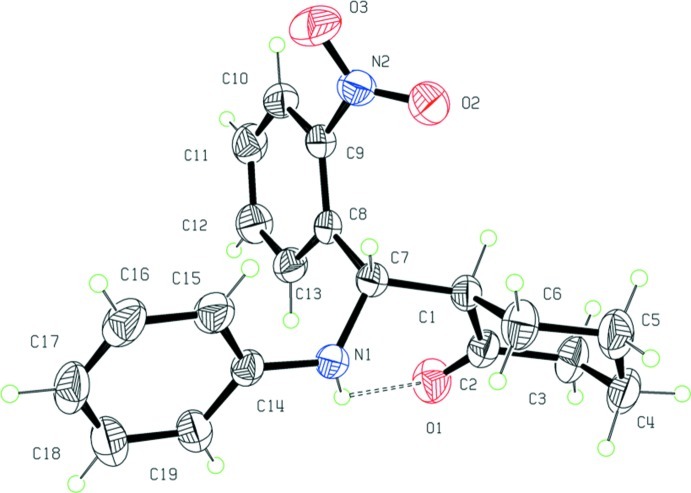
The structure of title compound, showing 35% probability displacement ellipsoids and the atom numbering scheme. Intramolecular hydrogen bond are shown as dashed lines.

**Fig. 2.**
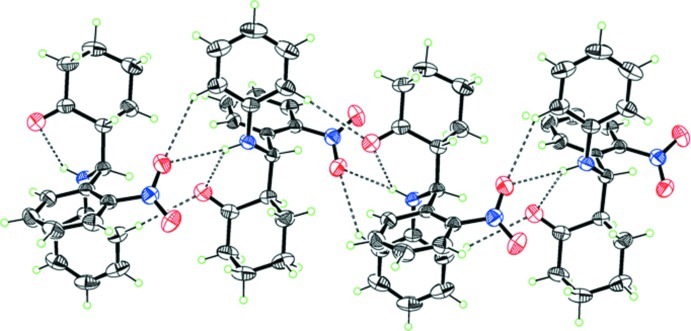
The stabilization of molecules in the crystal by inter- and intramolecular N—H···O hydrogen bonds and C—H···O interactions.

## supplementary crystallographic information

### Comment

*β*-amino ketones are widely found in natural and un-natural products, which exhibit anti-inflammatory (Jadhav *et al.*, 2008) and antimicrobial (Kalluraya *et al.*, 2001) activities. Mannich reaction (Eftekhari-Sis *et al.*, 2006; Samet *et al.*, 2009; Azizi *et al.*, 2006; Cordova, 2004) is one of the most important basic reactions in organic chemistry for its use in synthesis of *β*-Amino ketones. We have synthesized the title compound and report its structure here, Fig 1. The cyclohexanone ring adopts chair conformation, and aminomethyl moiety is positioned equatorially on ring at C1.

### Experimental

The title compound was obtained by adding of 0.04 g of Laponite-HMPC nano composite (Eftekhari-Sis *et al.*, 2012*a*,b) to a mixture of 0.5 mmol of 2-nitrobenzaldehyde, 0.5 mmol of aniline and 3 equiv. of cyclohexanone and stirring at room temperature for 24 h. After completion of the reaction, 5 ml EtOH was added and catalyst was removed by filtration, and filtrate was concentrated under reduced pressure. The obtained crud product was recrystallized from EtOH to afford title compound in 62% yield. Colorless crystals suitable for crystal structure determination were grown from EtOH.

### Refinement

Carbon bound H atoms were positioned geometrically, with C—H=0.93, 0.97, and 0.98 Å for aromatic, methylene and methine H, and constrained to ride on their parent atoms, with *U*_iso_(H) = 1.2Ueq(C). The nitrogen H atoms were located from the difference Fourier map allowed to refine freely.

### Crystal data

**Table:**

C_19_H_20_N_2_O_3_	*F*(000) = 688
*M**_r_* = 324.37	*D*_x_ = 1.297 Mg m^−^^3^
Monoclinic, *P*2_1_/*c*	Mo *K*α radiation, λ = 0.71073 Å
Hall symbol: -P 2ybc	Cell parameters from 10888 reflections
*a* = 9.0535 (8) Å	θ = 1.7–28.1°
*b* = 11.9947 (7) Å	µ = 0.09 mm^−^^1^
*c* = 17.2267 (15) Å	*T* = 296 K
β = 117.355 (6)°	Prism, colorless
*V* = 1661.5 (2) Å^3^	0.62 × 0.43 × 0.21 mm
*Z* = 4	

### Data collection

**Table:**

Stoe IPDS 2 diffractometer	3434 independent reflections
Radiation source: fine-focus sealed tube	2210 reflections with *I* > 2σ(*I*)
Graphite monochromator	*R*_int_ = 0.043
rotation method scans	θ_max_ = 26.5°, θ_min_ = 2.5°
Absorption correction: integration (*X-RED32*; Stoe & Cie, 2002)	*h* = −11→11
*T*_min_ = 0.784, *T*_max_ = 0.958	*k* = −15→14
10888 measured reflections	*l* = −21→20

### Refinement

**Table:**

Refinement on *F*^2^	Primary atom site location: structure-invariant direct methods
Least-squares matrix: full	Secondary atom site location: difference Fourier map
*R*[*F*^2^ > 2σ(*F*^2^)] = 0.054	Hydrogen site location: inferred from neighbouring sites
*wR*(*F*^2^) = 0.160	H atoms treated by a mixture of independent and constrained refinement
*S* = 0.97	*w* = 1/[σ^2^(*F*_o_^2^) + (0.099*P*)^2^] where *P* = (*F*_o_^2^ + 2*F*_c_^2^)/3
3434 reflections	(Δ/σ)_max_ < 0.001
221 parameters	Δρ_max_ = 0.79 e Å^−^^3^
16 restraints	Δρ_min_ = −0.23 e Å^−^^3^

### Special details

**Table:**

Geometry. All e.s.d.'s (except the e.s.d. in the dihedral angle between two l.s. planes) are estimated using the full covariance matrix. The cell e.s.d.'s are taken into account individually in the estimation of e.s.d.'s in distances, angles and torsion angles; correlations between e.s.d.'s in cell parameters are only used when they are defined by crystal symmetry. An approximate (isotropic) treatment of cell e.s.d.'s is used for estimating e.s.d.'s involving l.s. planes.
Refinement. Refinement of *F*^2^ against ALL reflections. The weighted *R*-factor *wR* and goodness of fit *S* are based on *F*^2^, conventional *R*-factors *R* are based on *F*, with *F* set to zero for negative *F*^2^. The threshold expression of *F*^2^ > σ(*F*^2^) is used only for calculating *R*-factors(gt) *etc*. and is not relevant to the choice of reflections for refinement. *R*-factors based on *F*^2^ are statistically about twice as large as those based on *F*, and *R*- factors based on ALL data will be even larger.

### Fractional atomic coordinates and isotropic or equivalent isotropic displacement parameters (Å^2^)

**Table:**

	*x*	*y*	*z*	*U*_iso_*/*U*_eq_	
C1	0.3530 (2)	0.26145 (18)	0.22061 (12)	0.0453 (5)	
H1	0.2966	0.3031	0.2482	0.054*	
C2	0.2739 (2)	0.1473 (2)	0.19939 (13)	0.0505 (5)	
C3	0.0911 (3)	0.1470 (3)	0.13652 (16)	0.0690 (7)	
H3A	0.0301	0.1828	0.1635	0.083*	
H3B	0.0522	0.0707	0.1229	0.083*	
C4	0.0580 (3)	0.2080 (3)	0.05272 (15)	0.0731 (8)	
H4A	0.1080	0.1670	0.0223	0.088*	
H4B	−0.0610	0.2115	0.0149	0.088*	
C5	0.1274 (3)	0.3236 (3)	0.07141 (16)	0.0730 (8)	
H5A	0.0699	0.3667	0.0969	0.088*	
H5B	0.1089	0.3592	0.0172	0.088*	
C6	0.3130 (3)	0.3223 (2)	0.13399 (16)	0.0660 (7)	
H6A	0.3532	0.3984	0.1468	0.079*	
H6B	0.3713	0.2856	0.1059	0.079*	
C7	0.5407 (2)	0.26636 (18)	0.28430 (12)	0.0429 (5)	
H7	0.5759	0.3436	0.2840	0.052*	
C8	0.5807 (2)	0.23927 (17)	0.37935 (12)	0.0414 (4)	
C9	0.5888 (2)	0.31751 (17)	0.44110 (13)	0.0448 (5)	
C10	0.6289 (3)	0.2903 (2)	0.52741 (15)	0.0574 (6)	
H10	0.6344	0.3455	0.5666	0.069*	
C11	0.6599 (3)	0.1815 (2)	0.55354 (16)	0.0655 (7)	
H11	0.6837	0.1613	0.6102	0.079*	
C12	0.6555 (3)	0.1022 (2)	0.49480 (16)	0.0636 (6)	
H12	0.6772	0.0281	0.5123	0.076*	
C13	0.6193 (3)	0.13097 (19)	0.41035 (14)	0.0531 (5)	
H13	0.6209	0.0757	0.3729	0.064*	
C14	0.8043 (2)	0.20265 (19)	0.28812 (12)	0.0455 (5)	
C15	0.8911 (3)	0.3024 (2)	0.31590 (16)	0.0613 (6)	
H15	0.8341	0.3686	0.3111	0.074*	
C16	1.0631 (3)	0.3029 (3)	0.35075 (17)	0.0752 (8)	
H16	1.1208	0.3698	0.3693	0.090*	
C17	1.1495 (3)	0.2056 (3)	0.35827 (16)	0.0750 (9)	
H17	1.2650	0.2063	0.3830	0.090*	
C18	1.0634 (3)	0.1077 (3)	0.32882 (17)	0.0726 (8)	
H18	1.1208	0.0422	0.3320	0.087*	
C19	0.8934 (3)	0.1054 (2)	0.29467 (15)	0.0580 (6)	
H19	0.8370	0.0381	0.2757	0.070*	
N1	0.6308 (2)	0.19843 (17)	0.25075 (11)	0.0467 (4)	
N2	0.5569 (2)	0.43539 (16)	0.41868 (13)	0.0545 (5)	
O1	0.3490 (2)	0.06234 (15)	0.22935 (12)	0.0718 (5)	
O2	0.4393 (2)	0.46052 (15)	0.34977 (12)	0.0711 (5)	
O3	0.6465 (3)	0.50448 (17)	0.47103 (14)	0.0861 (6)	
H1A	0.593 (3)	0.133 (2)	0.2363 (14)	0.046 (6)*	

### Atomic displacement parameters (Å^2^)

**Table:**

	*U*^11^	*U*^22^	*U*^33^	*U*^12^	*U*^13^	*U*^23^
C1	0.0372 (9)	0.0539 (13)	0.0416 (10)	0.0030 (8)	0.0155 (8)	0.0015 (9)
C2	0.0409 (10)	0.0643 (13)	0.0424 (11)	−0.0054 (8)	0.0158 (8)	0.0008 (9)
C3	0.0425 (11)	0.090 (2)	0.0587 (14)	−0.0105 (11)	0.0094 (10)	0.0033 (13)
C4	0.0445 (12)	0.115 (3)	0.0449 (12)	0.0038 (13)	0.0080 (10)	−0.0011 (13)
C5	0.0605 (14)	0.093 (2)	0.0534 (14)	0.0169 (13)	0.0157 (11)	0.0192 (13)
C6	0.0604 (13)	0.0680 (17)	0.0568 (14)	0.0042 (11)	0.0160 (11)	0.0186 (12)
C7	0.0384 (9)	0.0447 (11)	0.0438 (10)	−0.0013 (8)	0.0173 (8)	−0.0015 (9)
C8	0.0294 (8)	0.0471 (12)	0.0420 (10)	−0.0005 (7)	0.0116 (7)	−0.0023 (9)
C9	0.0356 (9)	0.0486 (12)	0.0474 (11)	0.0007 (8)	0.0166 (8)	−0.0039 (9)
C10	0.0526 (12)	0.0719 (17)	0.0474 (12)	0.0040 (10)	0.0226 (10)	−0.0071 (11)
C11	0.0619 (13)	0.086 (2)	0.0441 (11)	0.0112 (12)	0.0205 (10)	0.0105 (12)
C12	0.0692 (14)	0.0574 (16)	0.0569 (14)	0.0108 (11)	0.0227 (11)	0.0139 (12)
C13	0.0545 (11)	0.0482 (13)	0.0485 (11)	0.0039 (9)	0.0168 (9)	0.0007 (10)
C14	0.0387 (9)	0.0631 (14)	0.0350 (9)	−0.0016 (8)	0.0171 (8)	0.0002 (9)
C15	0.0499 (12)	0.0755 (17)	0.0601 (13)	−0.0119 (11)	0.0267 (10)	−0.0143 (12)
C16	0.0541 (14)	0.111 (2)	0.0618 (15)	−0.0289 (14)	0.0275 (12)	−0.0200 (15)
C17	0.0396 (11)	0.135 (3)	0.0495 (13)	−0.0052 (14)	0.0200 (10)	0.0048 (15)
C18	0.0518 (13)	0.104 (2)	0.0636 (15)	0.0196 (14)	0.0278 (11)	0.0205 (15)
C19	0.0481 (11)	0.0691 (16)	0.0570 (13)	0.0066 (10)	0.0243 (10)	0.0072 (11)
N1	0.0382 (8)	0.0505 (12)	0.0492 (10)	−0.0036 (7)	0.0184 (7)	−0.0092 (8)
N2	0.0535 (10)	0.0533 (12)	0.0605 (12)	−0.0005 (8)	0.0294 (9)	−0.0071 (9)
O1	0.0614 (10)	0.0610 (11)	0.0812 (12)	−0.0048 (7)	0.0226 (9)	0.0008 (9)
O2	0.0737 (11)	0.0559 (11)	0.0707 (11)	0.0117 (8)	0.0220 (9)	0.0046 (9)
O3	0.0958 (14)	0.0607 (12)	0.0903 (13)	−0.0195 (10)	0.0329 (11)	−0.0274 (10)

### Geometric parameters (Å, º)

**Table:**

C1—C2	1.510 (3)	C9—N2	1.459 (3)
C1—C7	1.543 (2)	C10—C11	1.367 (4)
C1—C6	1.547 (3)	C10—H10	0.9300
C1—H1	0.9800	C11—C12	1.376 (4)
C2—O1	1.202 (3)	C11—H11	0.9300
C2—C3	1.505 (3)	C12—C13	1.380 (3)
C3—C4	1.521 (4)	C12—H12	0.9300
C3—H3A	0.9700	C13—H13	0.9300
C3—H3B	0.9700	C14—C15	1.391 (3)
C4—C5	1.494 (4)	C14—C19	1.392 (3)
C4—H4A	0.9700	C14—N1	1.399 (2)
C4—H4B	0.9700	C15—C16	1.388 (3)
C5—C6	1.523 (3)	C15—H15	0.9300
C5—H5A	0.9700	C16—C17	1.378 (4)
C5—H5B	0.9700	C16—H16	0.9300
C6—H6A	0.9700	C17—C18	1.371 (4)
C6—H6B	0.9700	C17—H17	0.9300
C7—N1	1.448 (3)	C18—C19	1.373 (3)
C7—C8	1.541 (3)	C18—H18	0.9300
C7—H7	0.9800	C19—H19	0.9300
C8—C13	1.387 (3)	N1—H1A	0.85 (2)
C8—C9	1.395 (3)	N2—O2	1.214 (2)
C9—C10	1.398 (3)	N2—O3	1.219 (3)
			
C2—C1—C7	116.84 (17)	C9—C8—C7	124.96 (18)
C2—C1—C6	108.61 (18)	C8—C9—C10	123.4 (2)
C7—C1—C6	111.16 (16)	C8—C9—N2	121.02 (19)
C2—C1—H1	106.5	C10—C9—N2	115.56 (19)
C7—C1—H1	106.5	C11—C10—C9	119.2 (2)
C6—C1—H1	106.5	C11—C10—H10	120.4
O1—C2—C3	121.7 (2)	C9—C10—H10	120.4
O1—C2—C1	123.57 (18)	C10—C11—C12	119.0 (2)
C3—C2—C1	114.8 (2)	C10—C11—H11	120.5
C2—C3—C4	110.71 (19)	C12—C11—H11	120.5
C2—C3—H3A	109.5	C11—C12—C13	121.0 (2)
C4—C3—H3A	109.5	C11—C12—H12	119.5
C2—C3—H3B	109.5	C13—C12—H12	119.5
C4—C3—H3B	109.5	C12—C13—C8	122.4 (2)
H3A—C3—H3B	108.1	C12—C13—H13	118.8
C5—C4—C3	111.2 (2)	C8—C13—H13	118.8
C5—C4—H4A	109.4	C15—C14—C19	118.60 (19)
C3—C4—H4A	109.4	C15—C14—N1	121.8 (2)
C5—C4—H4B	109.4	C19—C14—N1	119.5 (2)
C3—C4—H4B	109.4	C16—C15—C14	119.8 (2)
H4A—C4—H4B	108.0	C16—C15—H15	120.1
C4—C5—C6	111.1 (2)	C14—C15—H15	120.1
C4—C5—H5A	109.4	C17—C16—C15	120.9 (3)
C6—C5—H5A	109.4	C17—C16—H16	119.6
C4—C5—H5B	109.4	C15—C16—H16	119.6
C6—C5—H5B	109.4	C18—C17—C16	119.3 (2)
H5A—C5—H5B	108.0	C18—C17—H17	120.4
C5—C6—C1	112.4 (2)	C16—C17—H17	120.4
C5—C6—H6A	109.1	C17—C18—C19	120.7 (3)
C1—C6—H6A	109.1	C17—C18—H18	119.6
C5—C6—H6B	109.1	C19—C18—H18	119.6
C1—C6—H6B	109.1	C18—C19—C14	120.7 (2)
H6A—C6—H6B	107.9	C18—C19—H19	119.6
N1—C7—C8	113.92 (16)	C14—C19—H19	119.6
N1—C7—C1	109.45 (16)	C14—N1—C7	121.03 (17)
C8—C7—C1	113.04 (15)	C14—N1—H1A	112.7 (14)
N1—C7—H7	106.6	C7—N1—H1A	114.4 (14)
C8—C7—H7	106.6	O2—N2—O3	122.8 (2)
C1—C7—H7	106.6	O2—N2—C9	118.52 (18)
C13—C8—C9	114.90 (19)	O3—N2—C9	118.7 (2)
C13—C8—C7	120.08 (18)		
			
C7—C1—C2—O1	0.1 (3)	C8—C9—C10—C11	−1.0 (3)
C6—C1—C2—O1	126.8 (2)	N2—C9—C10—C11	179.8 (2)
C7—C1—C2—C3	−179.56 (18)	C9—C10—C11—C12	1.9 (3)
C6—C1—C2—C3	−52.9 (2)	C10—C11—C12—C13	−0.4 (4)
O1—C2—C3—C4	−125.0 (3)	C11—C12—C13—C8	−2.1 (4)
C1—C2—C3—C4	54.7 (3)	C9—C8—C13—C12	2.9 (3)
C2—C3—C4—C5	−55.0 (3)	C7—C8—C13—C12	−179.79 (19)
C3—C4—C5—C6	56.5 (3)	C19—C14—C15—C16	−1.2 (3)
C4—C5—C6—C1	−56.3 (3)	N1—C14—C15—C16	−178.5 (2)
C2—C1—C6—C5	52.8 (3)	C14—C15—C16—C17	0.0 (4)
C7—C1—C6—C5	−177.3 (2)	C15—C16—C17—C18	1.6 (4)
C2—C1—C7—N1	56.4 (2)	C16—C17—C18—C19	−2.0 (4)
C6—C1—C7—N1	−69.0 (2)	C17—C18—C19—C14	0.8 (4)
C2—C1—C7—C8	−71.8 (2)	C15—C14—C19—C18	0.8 (3)
C6—C1—C7—C8	162.83 (19)	N1—C14—C19—C18	178.2 (2)
N1—C7—C8—C13	−32.7 (2)	C15—C14—N1—C7	−39.6 (3)
C1—C7—C8—C13	93.1 (2)	C19—C14—N1—C7	143.1 (2)
N1—C7—C8—C9	144.35 (18)	C8—C7—N1—C14	−63.0 (2)
C1—C7—C8—C9	−89.9 (2)	C1—C7—N1—C14	169.39 (18)
C13—C8—C9—C10	−1.4 (3)	C8—C9—N2—O2	44.2 (3)
C7—C8—C9—C10	−178.57 (17)	C10—C9—N2—O2	−136.5 (2)
C13—C8—C9—N2	177.87 (17)	C8—C9—N2—O3	−137.9 (2)
C7—C8—C9—N2	0.7 (3)	C10—C9—N2—O3	41.3 (3)

### Hydrogen-bond geometry (Å, º)

**Table:**

*D*—H···*A*	*D*—H	H···*A*	*D*···*A*	*D*—H···*A*
N1—H1*A*···O1	0.85 (2)	2.31 (2)	2.906 (3)	127.0 (18)
N1—H1*A*···O2^i^	0.85 (2)	2.48 (2)	3.246 (3)	150.2 (19)

Symmetry code: (i) −*x*+1, *y*−1/2, −*z*+1/2.
